# Novel coronavirus disease (COVID-19) pandemic: A recent mini review

**DOI:** 10.1016/j.csbj.2020.12.033

**Published:** 2020-12-31

**Authors:** Muhammad Fayyaz ur Rehman, Chaudhary Fariha, Aqsa Anwar, Naveed Shahzad, Munir Ahmad, Salma Mukhtar, Muhammad Farhan Ul Haque

**Affiliations:** a Institute of Chemistry, University of Sargodha, Sargodha 41600, Pakistan; b School of Biological Sciences, University of the Punjab, Lahore 54000, Pakistan

## Abstract

The COVID-19, caused by a novel coronavirus, was declared as a global pandemic by WHO more than five months ago, and we are still experiencing a state of global emergency. More than 74.30 million confirmed cases of the COVID-19 have been reported globally so far, with an average fatality rate of almost 3.0%. Seven different types of coronaviruses had been detected from humans; three of them have resulted in severe outbreaks, i.e., MERS-CoV, SARS-CoV, and SARS-CoV-2. Phylogenetic analysis of the genomes suggests that the possible occurrence of recombination between SARS-like-CoVs from pangolin and bat might have led to the origin of SARS-CoV-2 and the COVID-19 outbreak.

Coronaviruses are positive-sense, single-stranded RNA viruses and harbour a genome (30 kb) consisting of two terminal untranslated regions and twelve putative functional open reading frames (ORFs), encoding for non-structural and structural proteins. There are sixteen putative non-structural proteins, including proteases, RNA-dependent RNA polymerase, helicase, other proteins involved in the transcription and replication of SARS-CoV-2, and four structural proteins, including spike protein (S), envelope (E), membrane (M), and nucleocapsid (N). SARS-CoV-2 infection, with a heavy viral load in the body, destroys the human lungs through cytokine storm, especially in elderly persons and people with immunosuppressed disorders. A number of drugs have been repurposed and employed, but still, no specific antiviral medicine has been approved by the FDA to treat this disease. This review provides a current status of the COVID-19, epidemiology, an overview of phylogeny, mode of action, diagnosis, and possible treatment methods and vaccines.

## 1 Introduction

Coronaviruses (CoVs) belong to a large group of enveloped, single-stranded, positive-sense RNA viruses having the capability of infecting a wide variety of animals, including humans, birds, rodents, carnivores, chiropters and other mammals [1], [2]. Though they have been known for many years and have been considered as one of the viral sources responsible for respiratory diseases, they caught the attention of the whole world in December 2019, when an epidemic episode of cases with respiratory tract infections was reported in Wuhan, the largest metropolitan area in the province of Hubei, China. The outbreak was first treated as a complication of pneumonia with unknown etiology, but then the Centre for Disease Control in China declared that the respiratory infection was caused by a novel CoV named as 2019-nCoV, at that time [3], [4], [5], [6]. Later, the virus spread so enormously and rapidly that the WHO (World Health Organization) declared a global emergency amid this pandemic and called it coronavirus disease-2019 (COVID-19) while this novel 2019-nCoV was renamed as Severe Acute Respiratory Syndrome Coronavirus-2 (SARS-CoV-2). When the clinical spectrum of COVID-2019 was observed, it was noticed that few patients were asymptomatic, and some patients have mild to severe symptoms like severe respiratory discomfortness, fever, cough and flu [7], [8], [9]. During the past twelve months, COVID-19 has spread worldwide hitting some countries with extreme cruelty including the USA, India, Brazil, Russian Federation, France, the United Kingdom, Italy, Spain, Argentina, Colombia, Germany, Mexico, Poland, Iran, Turkey, each with more than 1 million confirmed COVID-19 cases (https://covid19.who.int/ accessed on December 19, 2020). Death toll has been extremely high in some countries including the USA, Brazil, India, Mexico, Italy, the UK, France, Iran, Russian Federation and Spain, each reporting greater than 50,000 COVID-19 related deaths as of December 19, 2020. According to WHO, 216 countries and territories around the world have reported more than 74.30 million confirmed COVID-19 cases with a death toll of above 1.67 million (https://covid19.who.int/ accessed on December 19, 2020).

Without any proper treatment and vaccine for COVID-19, we are currently experiencing a worldwide emergency affecting all societies, and it has sent billions of people into lockdown. Around the world, desperate efforts are underway to curtail this pandemic while it has resulted in the collapsing of health systems and has triggered lasting geopolitical and economic changes. To date, no approved medical treatment is available, that makes social distancing only best possible solution to stop the spread of the virus [10]. It is thought that future outbreaks of CoVs are unavoidable because of changes in the climate and ecology and increased interaction of humans with animals. Therefore, there is a need to develop effective therapeutics and vaccines against CoVs [11].

In this review, we briefly highlight the history, phylogeny, genomics, epidemiology, mode of action, disease symptoms, diagnosis, and possible treatment methods of COVID-19 and the research progress in the development of vaccines against SARS-Cov-2.

## 2 History of CoV-related diseases in humans

Human coronaviruses (HCoVs) were first reported in the mid-1960s when two species were isolated from persons with the common cold: HCoV-229E [12] and HCoV-OC43 [13]. Since then, seven different types of CoVs had been detected from humans*,* three of them happened to be highly pathogenic, and all suggested to be originated from bats: the Middle East respiratory syndrome coronavirus (MERS-CoV), severe acute respiratory syndrome coronavirus (SARS-CoV), and SARS-CoV-2 [14].

First time, CoV wreaked global havoc in 2002 when SARS-CoV caused a severe acute respiratory syndrome and emerged as highly pandemic disease. SARS-CoV was thought to be an animal virus with the genetic ability to cross the species barrier that spreads to humans through an unknown intermediate host(s) [4]. It first appeared as a human pathogen in the Guangdong province of southern China in 2002. Later, it spread to 26 countries and resulted in more than 8000 cases and 774 deaths in 2003 (http://www.who.int/csr/sars/country/table2004_04_21/en/). World Health Organization declared the end of this outbreak in July 2003.

Another respiratory syndrome outbreak similar to that of SARS-CoV emerged in June 2012 in Saudi Arabia and was named as MERS-CoV [15]. MERS-CoV outbreak infected 2494 individuals exclusively travelling through the Middle East and caused 858 deaths [16]. This virus originated from bats and possibly camels as its intermediate host, got passed genetic recombinations across different species to infect human beings [14].

A few months ago, a novel CoV emerged and caused a serious disaster across the whole world. During the last two months of 2019, several cases of ‘viral pneumonia’ in Wuhan, People’s Republic of China, were reported [17], [18]. The cause of this infectious disease was identified as a natural virus of an animal origin with spillover infection potential [19]. It was traced that the geographical source of this virus was Huanan South China Seafood Market, but the actual animal source of this CoV was not known. It is now thought that this virus came from bats as their primary hosts, then it passed through one or multiple intermediate hosts, possibly including pangolins, to infect human beings [20]. International Committee on Taxonomy of Viruses (ICTV) announced SARS-CoV-2 as the name of the new virus on February 11, 2020, because of the genetic resemblance of the virus to the CoV responsible for the outbreak of 2003. Following guiding principles previously developed with the World Organization for Animal Health (OIE) and the Food and Agriculture Organization (FAO) of the United Nations, WHO named the disease “COVID-19” and announced it as a global pandemic on March 11, 2020.

## 3 Epidemiology

**Fig. 1:**
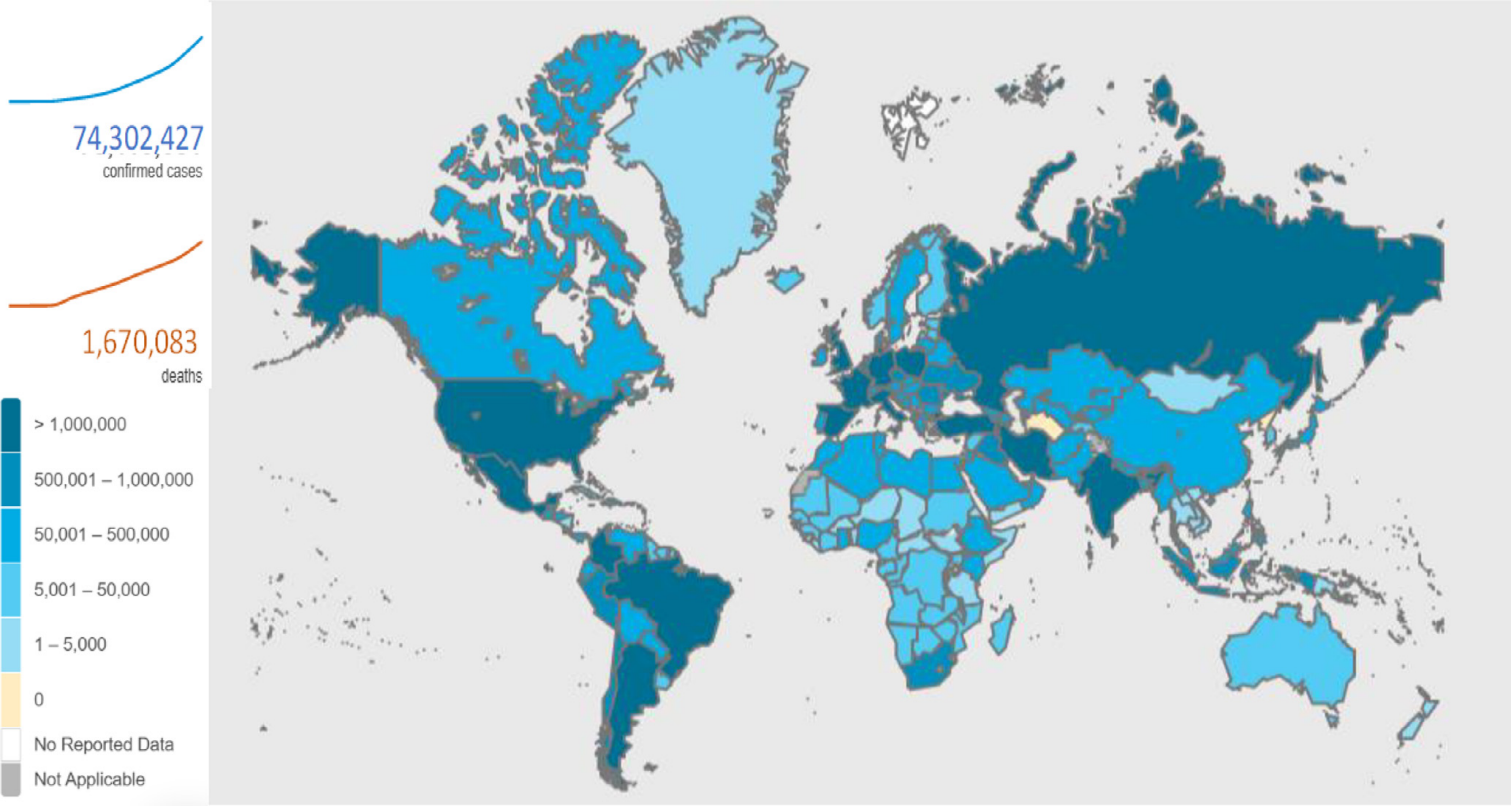
Total number of confirmed cases and deaths due to Coronavirus disease-2019 (COVID-19). Adapted from COVID-19 dashboard by WHO (https://covid19.who.int/) accessed on December 19, 2020.

Since the first confirmed diagnosis of SARS-CoV-2 in China, more than 74.30 million people have been affected, from which more than 1.67 million lives have been claimed (https://covid19.who.int/, assessed on December 19, 2020). Although more than 52 million people have defeated COVID-19 and recovered from the disease, yet the battle between SARS-CoV-2 and humans is continued, and still, no specific therapeutics are available. The United States of America (USA) shares 22.7% of total infection cases, followed by India and Brazil, sharing 13.5% and 9.6% of cases, respectively (https://covid19.who.int/, assessed on December 19, 2020) (Fig. 1). Although a decrease in death rate is observed (September 10, 3.22; July 20, 6.65%; April 10, 22.36% and Feb 2, 41.80%), there is no substantial reduction in active COVID-19 cases (>700,000daily cases on December 19, 2020). The cumulative incidences for COVID-19 vary by a multitude of factors, including comorbidities, age, gender, health and living conditions [21], [22]. The disease severity was found to increase in diabetes, cardiovascular, lung, kidney, and renal diseases [23]. Upon infection, one in five persons, with developed comorbidities, is at increased risk of severe COVID-19 infection [24]. Case studies from China show that COVID-19 is more severe in older adults aged 50–60 years [25], while it became more fatal in people above 70 years old regardless of any chronic disease complications. In a gender-based *meta*-analysis study of European countries, it is observed that COVID-19 was significantly fatal in men compared to women [26].

In the USA, the situation is still aggravating, where COVID-19 death toll is over 300,000 and the rate is still rising as 95 deaths per 100,000 since January 2020, across the country (https://www.cdc.gov/coronavirus/2019-ncov, accessed December 20, 2020). Amongst the countries reporting at least 50,000 COVID-19 cases, Singapore has the lowest COVID-19 fatality count with just 27 deaths with more than 57,000 persons who tested positive for COVID-19, with a death rate of below 0.05% compared to the global average of 3%. Singapore’s COVID-19 pandemic response that includes, mass testing, contact tracing of COVID-19 positive patient, rapid response public health preparedness clinics across the country, public awareness and countrywide lockdown [27] can be adapted as a successful model framework for other countries. In case no viable vaccine is available for low income countries, Africa, South Asia and South America can become unfortunate regions severely affected by SARS-CoV-2. A recent estimate put 23 million African population at the risk of severe COVID-19, whereas the current infection rate is exponentially increasing by 0.22 per day [28], [29]. Overloading poorly established health systems in underdeveloped countries may lead to numerous causalities.

## 4 Taxonomy and phylogeny of SARS-CoV-2

**Fig. 2:**
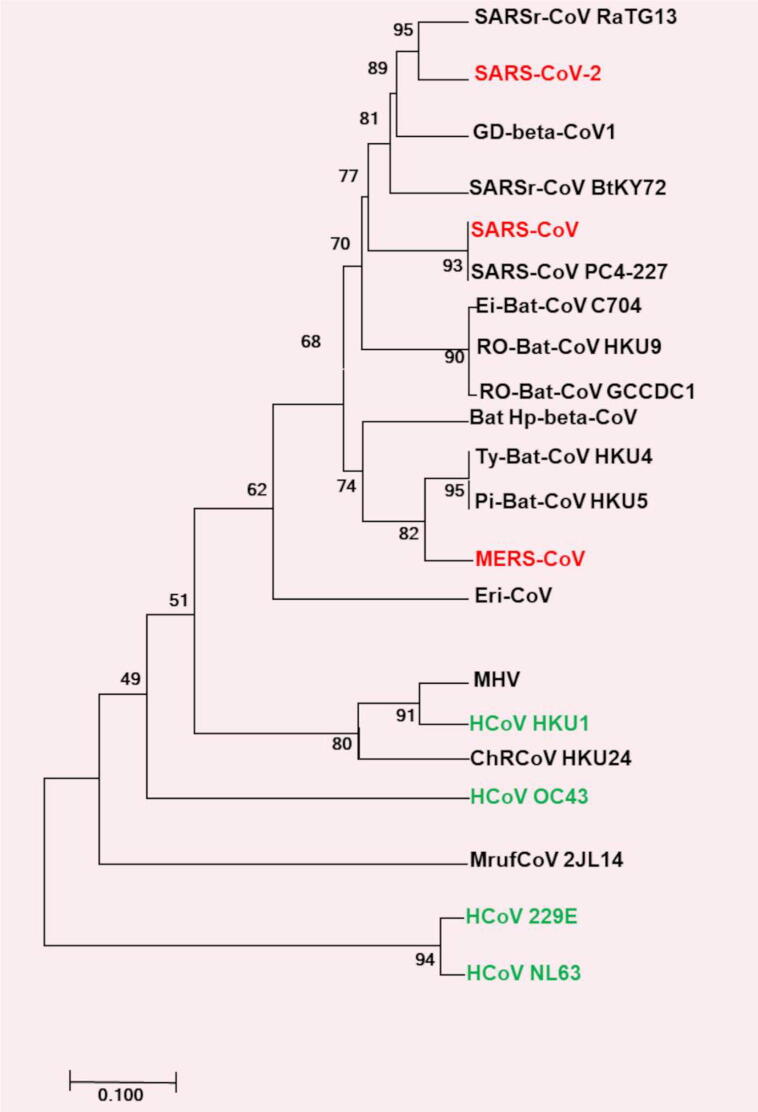
Phylogenetic tree of representative species of SARS-CoV-2, SARS-CoV, and MERS-CoV. Red text highlights zoonotic viruses with pathogenicity in humans and green text highlights common respiratory viruses that circulate in humans*.* The percentage of replicate trees in which the associated taxa clustered together in the bootstrap test (1,000 replicates) was shown next to the branches. Figure adapted from Gorbalenya et al., [30]. The tree was drawn based on the sequence information of following species: Severe acute respiratory syndrome related Bat Hp-betacoronavirus Zhejiang2013 (SARSr-CoV Ratg13), Rousettus bat coronavirus GCCDC1 (RO-Bat-CoV GCCDC1), Rousettus bat coronavirus HKU9 (RO-Bat-CoV HKU9), Eidolon bat coronavirus C704 (Ei-Bat-CoV C704), Pipistrellus bat coronavirus HKU5 (Pi-Bat-CoV HKU5), Tylonycteris bat coronavirus HKU4 (Ty-Bat-CoV HKU4), Middle East respiratory syndrome-related coronavirus (MERS-CoV), Hedgehog coronavirus OC43 (HCoV OC43), Murine coronavirus (MHV), Human coronavirus HKU1 (HCoV HKU1), China Rattus coronavirus HKU24 (ChRCoV HKU24), Pangolin Beta-coronavirus (GD-beta-CoV1), Bat Betacoronavirus 1 (Bat Hp-beta-CoV1), Myodes coronavirus 2JL14 (MrufCoV 2JL14), Human coronavirus NL63 (HCoV NL63), Human coronavirus 229E (HCoV 229E). (For interpretation of the references to colour in this figure legend, the reader is referred to the web version of this article.)

CoVs are positive-sense, single-stranded RNA viruses belonging to the order Nidovirales, suborder Cornidovirineae, family Coronaviridae and subfamily Orthocoronavirinae [16], [30]. The subfamily Orthocoronavirinae is further divided into Alpha-, Beta-, Gamma- and Delta- CoVs [31]. Alpha- and Beta- CoVs are pathogenic to mammals, including human beings, bats, pigs, mice, and cats. Gamma- and Delta- CoVs are usually pathogenic to birds but rarely infectious to mammals [32]. Phylogenetic analysis of SARS-CoV-2, SARS-CoV, and MERS-CoV suggests that it is more closely related to bat-CoVs of the Sarbecovirus subgenus isolated from bats (Fig. 2). A SARS related (SARSr) bat-CoV strain named SARSr-CoV-RaTG13 detected in an Intermediate Horseshoe bat (*Rhinolophus affinis*) [33], [34], was found very similar to SARS-CoV-2. The comparison of genome sequences revealed that SARSr-CoV-RaTG13 and SARS-CoV-2 sequences shared a similarity of more than 96% over a large part of the genome. However, the genomic region spanning the 3′-end of ORF1a, ORF1b and almost half of the spike (S) protein region of SARS-CoV-2 is divergent to SARSr-CoV-RaTG13 [35] but more closely related to pangolin CoV [36]. Considering that bats were in hibernation when the outbreak occurred [37], and the phylogenetic resemblance of Pangolin CoV strain to SARS-CoV-2, suggest that the virus is more likely to have been transmitted via other species. This also suggests that the possible occurrence of recombination between SARS-like-CoVs from pangolin and bats might have led to the origin of SARS-CoV-2 [36] and the COVID-19 outbreak. Dorp et al., (2020) analyzed the emergence of genomic diversity over time and reported that all CoV sequences share a common ancestor towards the end of 2019, supporting this as the period when SARS-CoV-2 jumped into its human host. They further identified several recurrent mutations producing non-synonymous changes in the virus at the protein level, suggesting possible ongoing adaptation of SARS-CoV-2 to the human host [38]. Various sequencing projects and phylogenetic studies involving SARS-CoV-2 genomes from COVID-19 patients during this pandemic have revealed that how fast the virus is mutating and adapting to its novel human host, providing information to direct drug and vaccine design [39], [40], [41].

## 5 Genomic features and life cycle of SARS-CoV-2

**Fig. 3:**
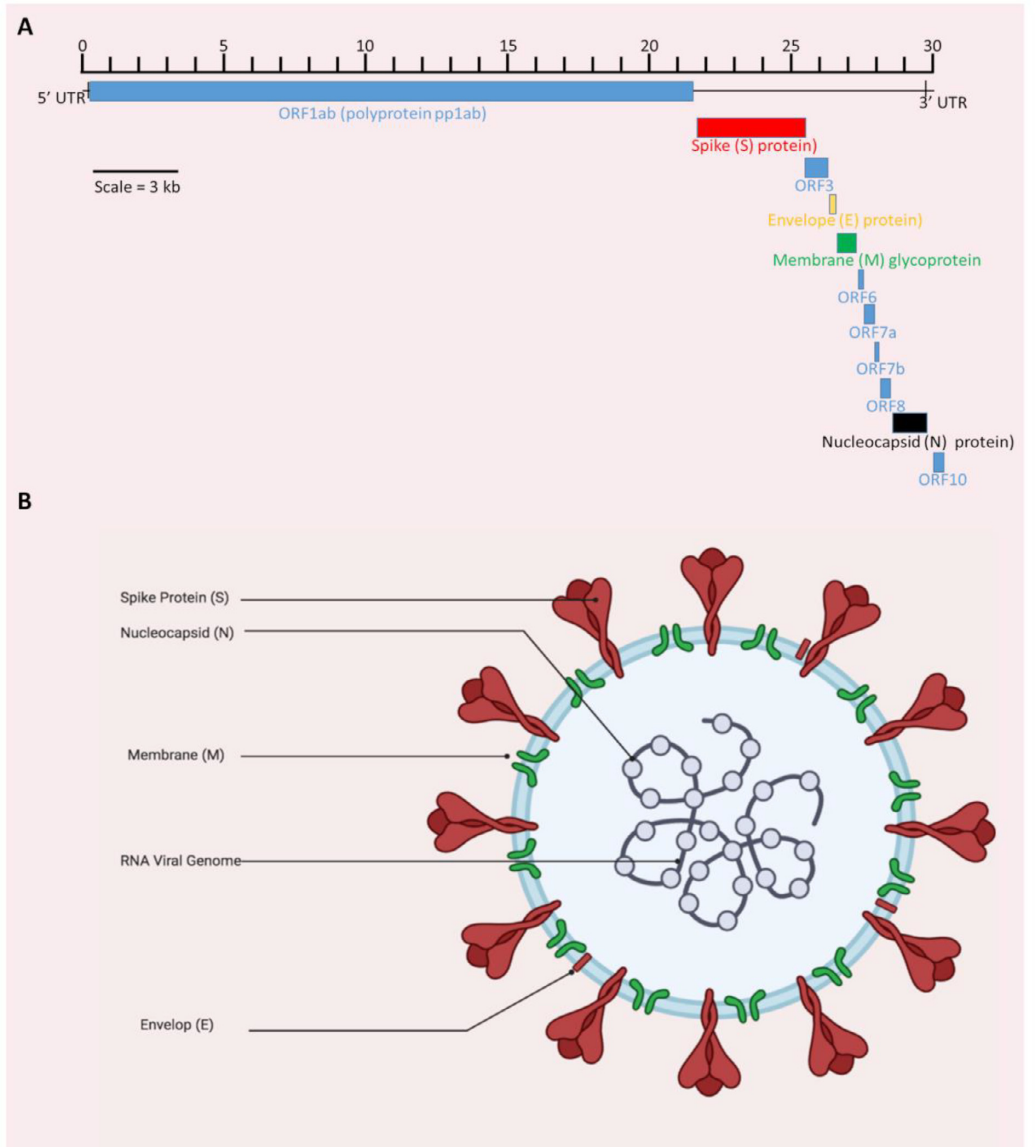
Genomic features and structure of SARS-CoV-2. **(A)** Genomic organization of SARS-CoV-2 reference genome (isolate Wuhan-Hu-1) from NCBI (accession number NC_045512.2). All genomic regions or open-reading frames (ORFs) are presented i.e. untranslated regions at both 5′ and 3′ ends (5′-UTR, 3′-UTR), polyproteins (pp1ab), structural proteins including spike (S), envelope (E), membrane (M) and nucleocapsid (N) proteins. **(B)** Structure (created with BioRender.com) of SARS-CoV-2 showing its major structural proteins.

Several genome sequences of SARS-CoV-2 retrieved from the COVID-19 patients have been reported by researchers from various countries. According to the NCBI database, there are more than 28,000 (full length) SARS-CoV-2 genome sequences from human hosts and more than 113,000 Sequence Read Archive (SRA) high throughput sequence submissions through multiple cloud providers and NCBI servers (December 20, 2020). The single-stranded positive-sense RNA genome of SARS-CoV-2 is around ~ 30 kb that starts with a 5′‐cap structure and ends with a 3′‐poly‐A tail. Chan et al. (2020) [42] reported detailed genomic characterization of SARS-CoV-2, which consists of two terminal untranslated regions (5′- and 3′- UTRs) and twelve putative functional open reading frames (ORFs) (Fig. 3**A**). ORFs 1a and 1b, spanning over two-thirds of the genome, encode the large replicase polyproteins (pp1a and pp1ab), which are post-translationally cleaved into 16 putative non-structural proteins (nsps) involving proteases, RNA polymerase, helicase, and other proteins involved in the transcription and replication of SARS-CoV-2 [4], [11], [42]. There are four structural proteins, including S protein, envelope (E), membrane (M), and nucleocapsid (N) (Fig. 3**B**) [43]. Most of the nonstructural proteins are known to have a role in the replication of the viral genome, whereas these four structural proteins are essential for the assembly and release of SARS-CoV-2 [43]. S protein is responsible for the binding of virion on the cell surface [44]. M protein has three transmembrane domains, whereas E protein plays its role in the assembly and release of viral particles from the cell. It is also involved in viral pathogenesis [45]. N protein has two domains, both of which can attach to the viral RNA in order to assist replication, and it also acts as a repressor of the RNAi system of the host cell, hence supporting the viral replication [46]

The initial attachment of the virus to the host cell is mediated by S protein, which has two subunits, S1 (specific receptor binding domain known as RBD) and S2 (CoV S2 glycoprotein). S protein, through its specific RBD, binds to its receptor on the host cell [47]. Wan et al. [48] reported that SARS-CoV-2 is optimized to bind on angiotensin-converting enzyme II (ACE2) human receptors. RBD in the S protein is the most variable part, and it differs for each type of CoV. After binding to the receptor, the host protease cleaves the S protein, which causes the release of the spike fusion peptide, facilitating the entry of the virus into the host cell [49]. S protein cleavage takes place at two sites. The first cleavage causes the separation of RBD and fusion peptide, whereas the second cleavage exposes the fusion peptide that inserts into the cell membrane [50], which ultimately causes the formation of a six-helix bundle. The formation of this bundle allows the fusion of virus cell membrane and host cell membrane, causing the release of the viral genome into the cytoplasm [51].

As the genome of CoV consists of positive-sense single-stranded RNA, it is used as a template directly to translate pp1a and pp1b, which are processed further to proteins essential for the formation of replication transcription complex (RTC) present in double-membrane vesicles [52]. Subsequently, RTC synthesizes a set of sub-genomic RNAs (sgRNAs) in a discontinuous manner [53]. The positive sgRNA serves as an mRNA for all structural and accessory genes, whereas the negative-sense strand of sgRNA serves as a template for the production of sub genomic and genomic positive-sense mRNAs [54]. Following the replication and synthesis of mRNAs, structural proteins get transcribed [55]. These structural proteins are inserted into the endoplasmic reticulum and transferred to endoplasmic reticulum-Golgi intermediate compartments [56]. Here, the genomes are encapsulated by N proteins and budded into membranes of ERGIC containing viral structural proteins, ultimately causing the release of the mature virion. This causes an increase in the viral load in the body.

## 6 Pathogenesis of SARS-CoV-2

**Fig. 4:**
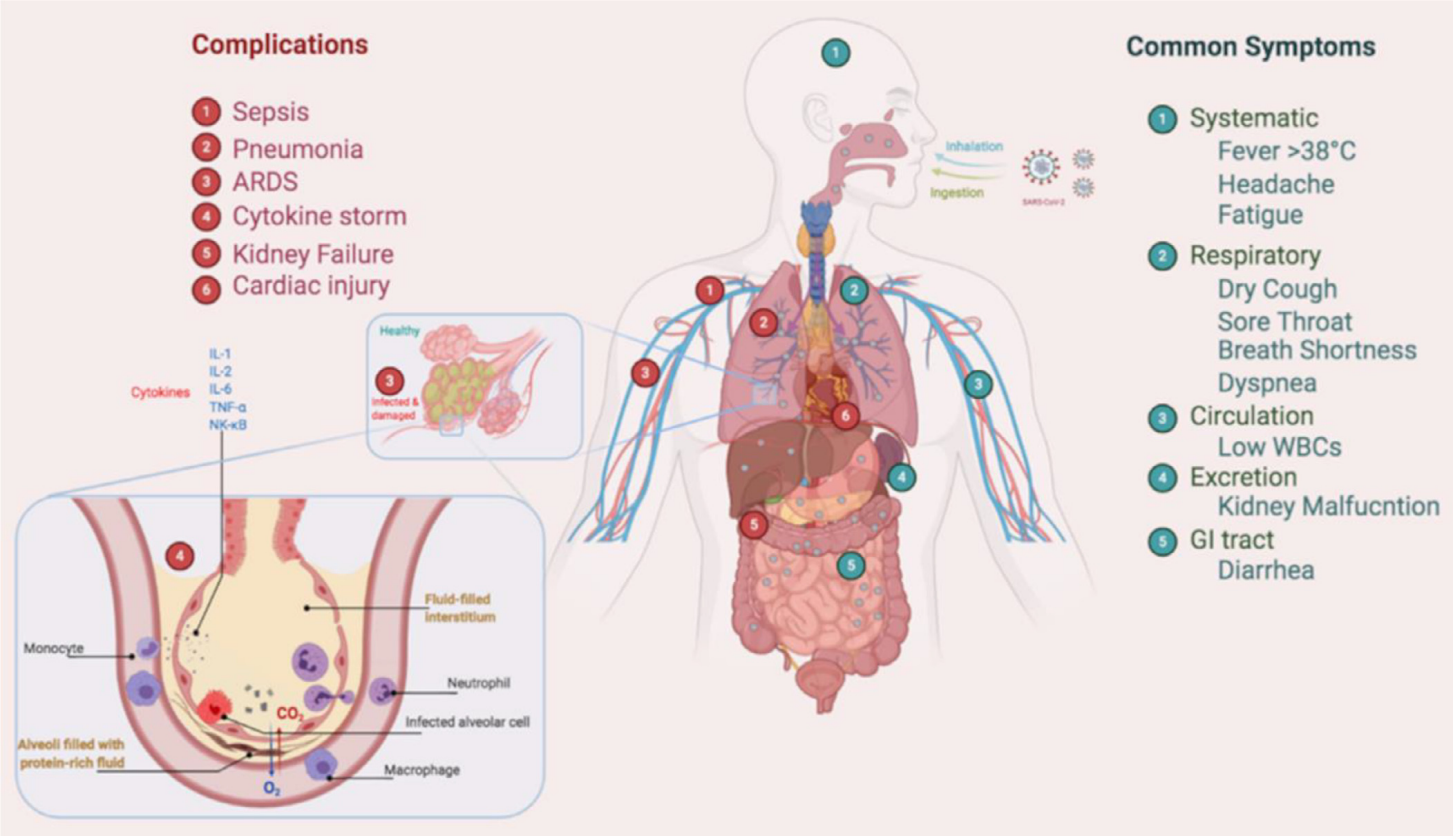
Common symptoms and complication related to the patients of coronavirus disease-2019 (COVID-19). (Figure created with BioRender.com).

SARS-CoV-2 manipulates host’s receptor ACE-2 and a serine protease TMPRSS2, to activate viral S protein and entry inside the host cell [57], [58], [59]. SARS-CoV-2 infection, with heavy viral load in the body, destroys the human lungs through cytokine storm that refers to the overreaction of the body’s immune system [60]. Cytokines released by different types of cells in the body, are signals to attract immune cells to the site of infection, which allows the immune cells to coordinate their response against the virus. During a viral infection, the body produces large amounts of cytokines, causing a significant burden on the immune system, referred to as cytokine storm syndrome [61]. This burden forces the immune system to send more and more immune cells to the site of infection, leading to hyper-inflammation (Fig. 4). CoVs, after entering into the lungs, reaches the lower respiratory tract where alveoli are present and start to replicate there [62]. As a result, the cytokine storm causes the destruction of alveoli by the immune system. More and more immune cells are recruited to the site of infection that leads to the thickening of the lung lining and ultimately causes pneumonia with shortness of breath, the main symptom of COVID-19 [62]. Moreover, this cytokine storm forces the immune cells to destroy the healthy cell lining of the lungs that may leads to secondary bacterial pneumonia, causing the lungs to become less functional. Owing to the malfunctioning of lungs, other organs such as the brain, kidney, and liver become deprived of oxygen. Eventually, patients require ventilators to receive enough oxygen [63].

## 7 Symptoms

The effect of COVID-19 may vary from person to person, and it may be from mild to moderate with an incubation period of 6 to 41 days (median of 14 days) [64]. The manifestation of multiple COVID-19 symptoms, as well as the duration of incubation time, depends on age groups, health conditions, and exposure times [65]. Old age people and patients with immunosuppressed disorders are the most susceptible to the infection. On average, symptoms appear in 5 days after exposure [66]. These symptoms may range from headache, fatigue with pain and aches, cough, sore throat to high fever, GI distress, diarrhea, nausea, myalgia, dyspnea, lymphopenia, difficulty in breathing and pneumonia [67], [68], [69], [70]. Unfortunately, COVID-19 symptoms, at the initial stage cannot make the basis for diagnosis as they mimic many respiratory and common infections (Table 1). Moreover, SARS-CoV-2 infected persons might also be asymptomatic carriers.

**Table 1:** Characteristics, symptoms and epidemiology of respiratory viruses.

COVID-19 may progress with cytokine storm leading to acute pneumonia, acute respiratory distress syndrome (ARDS) (Fig. 4), respiratory failure and even death [71]. Acute lung and kidney injuries, shock and heart failure have also been observed as complications of the disease [69], [70], [71]. Some individuals fail to respire in the fulminant disease that causes septic shock, multiple organ dysfunction (MOD), multiple organs failure (MOF), and its frequency is 5% patients [69], [70], [71]. Chest radiographs from COVID-19 patients indicate pneumonia with peripheral and subpleural ground-glass lung opacities [72], [73]. The proinflammation state with cytokine storm and an imbalance expression of ACE receptors are associated with the progression of COVID-19 [74]. SARS-CoV-2 interacts with sialic acids present at the surface of ACE2 receptors [74], reduces their expression, and triggers proinflammatory mediators, including IL-6, IL2, NF-κB, and TNF-alpha [75], [76].

## 8 Diagnostics

Unbiased next-generation sequencing tools were used for the confirmed diagnosis of earlier cases after initial screening for common causes of respiratory infections of patients with atypical pneumonia due to an unidentified microbial agent gave negative results [77], [78]. Next-generation sequencing of bronchoalveolar lavage was performed, and a novel CoV later named SARS-CoV-2 was subsequently identified as the causative pathogen [78], [79]. Few weeks following the preliminary characterization of COVID-19, molecular assays for detection of the virus in clinical samples were rapidly developed using the sequenced genomic information. Corman et al., developed a diagnostic qRT-PCR-based protocol for COVID-19 using swabbed samples from a patient’s nose and throat that has since been selected by the World Health Organization (https://www.who.int/docs/default-source/coronaviruse/protocol-v2-1.pdf?sfvrsn = a9ef618c_2). The Chinese and American Centers for Disease Control and Prevention and other research groups also described the development of real-time PCR methods to diagnose COVID-19, mainly targeting various combinations of the ORF1ab, E, N, and RNA-dependent RNA polymerase (RdRp) genes [80], [81], [82], [83]. Few cases of the COVID-19 reinfection have also been reported based on re-positive PCR test [84]. Though few studies have suggested short lived immunity to be a reason of reinfection [85], while some reports suggests the involvement of false positives, wrong sampling and medical diagnosis [86].

Although qRT-PCR has been a gold standard for the diagnostic of COVID-19 and the detection of SARS-CoV-2, several other methods have also been developed. A new molecular approach for the diagnosis of COVID-19 is Loop-mediated isothermal amplification (LAMP) being emerged as a great alternative to the qRT-PCR method. Amplification at a constant temperature (60 to 65 ͦC), exclusion of fancy lab instruments, rapid test results, naked-eye visible results, and potentially a numerically large diagnostic capacity, while maintaining similar sensitivity and specificity [87] are the advantages LAMP assay possesses thus making it more suitable than the RT-PCR during a pandemic period. LAMP assay is relatively a newer technique and therefore, there is less evidence on its use, but several studies have reported the development of LAMP assays for the detection of SARS-CoV-2 in clinical [88], [89], [90], [91] as well as environmental samples (Farhan Ul Haque et al., unpublished). Triggering the neutralizing antibody response to CoV infections [68] also allowed the development of serological testing. Several serodiagnostic methods have been developed for the rapid detection of COVID-19 [92], [93], [94], [95], [96], [97], but some of these methods have been reported inadequate in clinical settings due to very low sensitivity [98].

## 9 Treatments and vaccines

**Fig. 5:**
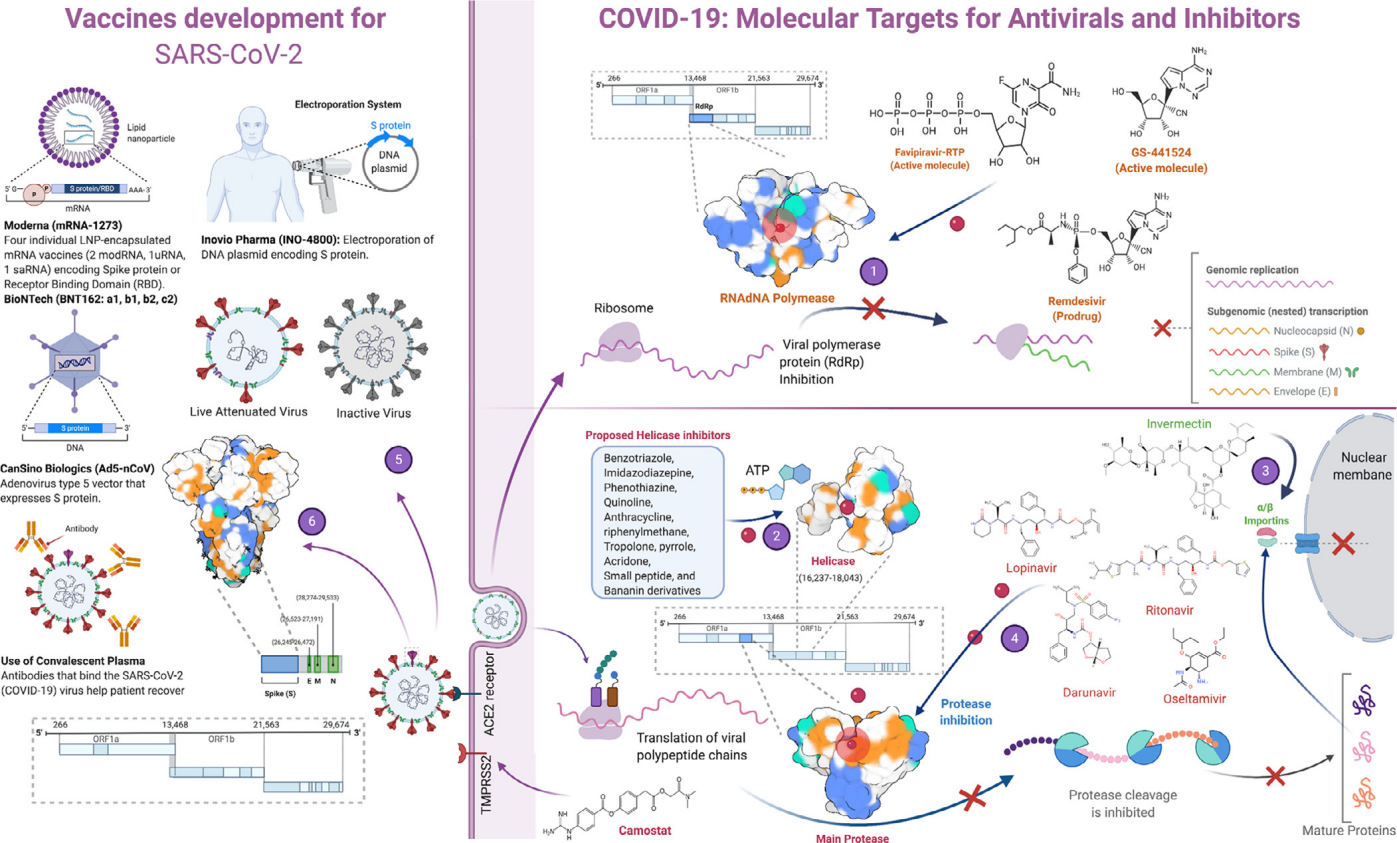
Development of repurposed drugs and vaccines against COVID-19. (1) Inhibition of RNA-dependent RNA polymerase by Favipiravir, Remdesivir and GS-441524. Inhibition of the enzyme halts genomic replication and stops viral dissemination (2) Several drugs have been proposed against helicase but none of them is approved yet (3) Ivermectin dissociates IMP α/β1 (importins) heterodimer, which is responsible for nuclear transport of viral protein cargos, so viral proteins cannot enter into the nucleus to continue vital processes like replication (4) Lopinavir, Ritonavir, Darunavir and Oseltamivir inhibit Main Protease (M^Pro^) enzyme which is involved in maturation of viral proteins (5) Inactive and attenuated SARS-CoV-2 can be used for vaccine production (6) Use of spike protein for development of vaccine candidates by different labs and pharma companies. (Figure created with BioRender.com).

A number of drugs have been repurposed and employed (Fig. 5) for COVID-19 treatment [99] but still, no specific and effective drug has been approved to treat this disease. By 3rd September 2020, 321 vaccine candidates had been proposed and 33 of them were in clinical trials [100]. Along with traditional therapeutics, monoclonal antibodies, [101], [102], [103] convalescent blood plasma, [104], [105] peptide-based [106] and oligonucleotide medicines, [107] and interferon therapies (Inhaled interferon beta) [108], [109] have been used to treat COVID-19. As 80% of the COVID-19 victims suffer from mild symptoms, they do not need any special medical care. The best treatment for those people is to self-isolate themselves along with a healthy diet. Old-age and patients with comorbidities are required to be admitted to the hospital and sometimes may need oxygen and ventilator support [9].

During the early days of the COVID-19 pandemic, synthetic forms of quinine, chloroquine (CQ), and hydroxychloroquine (HCQ) were proposed as medicine of choice against COVID-19. In contrast, HCQ was previously found potent against several viral diseases including avian influenza A H5N1, HIV-1/AIDS, hepatitis C virus, Dengue virus, Zika virus, Ebola virus and SARS [110], [111], [112], [113], [114], [115], [116], [117]. These drugs have been proposed to inhibit posttranslational modifications in ACE2 receptors in humans and interfere with SARS-CoV-2 interactions with cells. HCQ, in combination with a macrolide antibiotic, azithromycin, was widely proposed as COVID-19 treatment, but the recent metanalyses show the combination of two medicines increased the mortality rate in the treated patients [118], [119]. Later, a debate was started for CQ and HCQ use against SARS-CoV-2 as some clinical trials reported adverse events during the treatment, including arrhythmia, heart failure and increased death rate [120], [121]. World Health Organization (WHO) and the National Institute of Health (NIH) stopped many trials using HCQ treatments afterwards and FDA revoked the use of HCQ and CQ for COVID-19 treatment [122]. Moreover, the “Randomised Evaluation of COVid-19 thERapY (RECOVERY)” trial conclusively showed that hydroxychloroquine is not an effective treatment in patients hospitalized with COVID-19. The RECOVERY trial was established during March 2020 as a randomized clinical trial in the UK to test a range of potential drugs for COVID-19 cure (https://www.recoverytrial.net/). The trial is still going on and several drugs or treatments including Colchicine, Dexamethasone, Lopinavir-Ritonavir, Azithromycin, Tocilizumab, Convalescent plasma (collected from donors who have recovered from COVID-19 and contain antibodies against SARS-CoV-2) and Aspirin, have been repurposed in an attempt to find an effective cure for COVID-19 patients. Montelukast, a cysteinyl leukotriene receptor antagonist used to treat asthmatic attacks, has been found better than HCQ in a randomized observational study, where it not only tames the cytokine storm in severe COVID-19 patients but also decreases the duration of the disease (Rehman et al., unpublished).

Antiviral drugs, including Galidesivir, Favipiravir, Remdesivir, Lopinavir/Ritonavir, Umifenovir (Arbidol), Ostalmovir have also been found active against SARS-CoV-2 [123], [124]. Galidesivir, Favipiravir and Remdesivir are nucleoside analogues and inhibit viral RNA-dependent RNA polymerase [123], [124]. Galidesivir has been previously used against the Ebola virus, HCV and Marburg virus [125]. Favipiravir is an antiviral effective against the influenza virus and in recent clinical trials, Favipiravir was found useful for COVID-19 [126]. Remdesivir, a non-FDA-approved antiviral was previously found active against SARS-CoV and MERS-CoV. It has also shown promising effects against SARS-CoV-2, and the FDA has allowed the emergency use of the drug to treat severe COVID-19 patients on 1 May 2020. Lopinavir/Ritonavir is a protease enzyme inhibitor, Ostalmovir is a Neuraminidase inhibitor, and Umifenovir interferes with virus interactions with host cells [123]. All of these antivirals have been employed in many clinical trials and found effective against COVID-19 to some extent [127], [128], [129].

Nutraceuticals, food supplements and phytochemicals have shown great potential to fight against many deadly viral diseases including SARS-CoV, HIV, HBV, HCV and Dengue fever [130], [131], [132]. Several plant-derived constituents including the polyphenols, alkaloids, terpenoids have been studied as potential inhibitors of SARS-CoV-2 surface protein (S protein) [133], [134] and key enzymes i.e., proteases, helicase, and polymerase [135], [136], [137]. *In silico* approaches have found more than 100 phytoconstituents including hesperidin, rhodiolin, baicalin, glycyrrhizin, and 18, ß-Glycyrrhetinic acid to interact with SARS-CoV-2 enzymes/proteins with high binding affinity [137], [138] (Rehman et al., unpublished) where as these phytochemical have already been experimentally found active against many viral enzymes [139], [140], [141]. Some traditional Chinese medicines (TCM) and phytoextracts are also being used in clinical trials against SARS-CoV-2 [142], [143].

A number of monoclonal antibodies act against inflammatory cytokines. Tocilizumab, a recombinant humanised monoclonal antibody (Anti IL-6), is conventionally used to treat rheumatoid arthritis and has shown promising effects in taming the cytokine storm in severe COVID-19 patients [79], [144]. Sarilumab is another antagonist to the IL-6 receptor that is undergoing phase 2/3 trials for the treatment of COVID-19 [145]. Camostat mesylate is usually used for the treatment of pancreatitis, is approved to be effective against SARS-CoV-2 by preventing its entry into the host cell [146]. For the treatment of COVID-19, α, and β- interferon therapy was found very useful, especially when used in combination with other drugs like lopinavir or ribavirin. But like the number of other factors, delay in treatment reduces the effectiveness of interferon [147]. The use of interferons is not recommended for the treatment of COVID-19. Treatment of severe COVID-19 patients using convalescent plasma or immunoglobulins from the recovered patients has also been found successful against SARS-CoV-2 [148], [149]. Corticosteroids can help in alleviating lung inflammation, but their uses may lead to other complications like hyperglycemia, avascular necrosis, and psychosis [150]. The use of dexamethasone has been found safer to lower down the mortality rate in COVID-19 patients [151].

According to WHO, till the end of November 2020, almost a year into this on-going pandemic, there was no FDA-approved vaccine available against COVID-19. Considering the severity of the current public health emergency worldwide, FDA has issued an emergency use authorization for two vaccines: mRNA-1273 vaccine from Moderna and BNT162b2 from Pfizer – BioNtech. Apart from these two vaccines, currently 172 countries are working on the development of an efficient and safe vaccine [152]. S protein of SARS-CoV-2 has been targeted for the development of the majority of vaccines [153], [154]. Microneedle array delivered recombinant CoV vaccine, PittCoVacc, has been proposed to develop immunity in mice against the CoV within just two weeks of microneedle pricks [155]. Lab mice produced specific antibodies in amounts sufficient to neutralize the virus. The vaccine was delivered via a fingertip-sized patch of 400 tiny needles, which are designed to provide the S protein pieces directly into the skin, where the immune system is strongest. Bacillus Calmette-Guerin (BCG) is also another potential candidate to fight against COVID-19 that offers a boarder protection against various respiratory infections [156]. Countries that have a late start of universal BCG vaccine policy also had a high mortality rate, supporting the idea that BCG protects the vaccinated population from SARS-CoV-2 [156], [157].

Inovio, a Pennsylvania-based biotech company, is using DNA instead of RNA for making the candidate vaccine (INO-4800) against the COVID-19 [158]. Zydus Cadlia, an India-based pharmaceutical company, is using two approaches for the development of the COVID-19 vaccine [18]. First, is the use of DNA to produce CoV protein in the human body and second deals with the genetically manipulating an attenuated measles virus to boost the immune response against COVID-19. Novavax, a Maryland-based company, announced that they had generated a candidate vaccine using recombinant proteins nanoparticles derived from the S proteins of SARS-CoV-2 in February [159]. Altimmune is developing a candidate vaccine that gets sprayed into patient’s noses instead of injecting them into their arms [160]. Vaxart is developing an oral vaccine against COVID-19, whereas another company, Expression, is using insect cells from fruit fly to produce viral antigens [161]. Many other pharmaceutical companies are using different approaches to develop candidate vaccines, but Codagenix is the only company to attenuate a live SARS-CoV-2 virus to develop a vaccine [162]. They are using the deoptimization approach to manipulate the virus in such a way that it may replicate inside both without causing any disease. COVAX (COVID-19 Vaccines Global Access) COVAX has the largest vaccine portfolio for the COVID-19 [163]. They have nine candidate vaccines and nine more vaccines under consideration. COVAX is co-led by CEPI (The Coalition for Epidemic Preparedness Innovations), ACT (Access to COVID-19 Tools), WHO, the vaccine Alliance and Gavi. One vaccine is approved by the Ministry of Health of the Russian Federation on 11 August. The name of the vaccine is Sputnik-V, previously known as COVID-Vac [164]. The Gamaleya Research institute developed this vaccine in Moscow. Medicago is going to develop a vaccine that is a SARS-CoV-2 like particle. They proposed that this virus-like particle would force the immune system of humans to produce antibodies against SARS-CoV-2. Overall, without approved and specific anti-SARS-CoV-2 drugs and vaccines, it is very difficult to treat the patients with severe COVID-19, so currently, the main focus during the treatment of COVID-19 patients is to maintain the functions of patients’ organs.

Nano-formulations of the proposed therapeutics and vaccines can target the effected cells through inhalation or intravenous injection in a way with better efficiency and efficacy [165]. Nano-antibodies (nanobodies) have been developed to treat COVID-19 patients. Nanodrugs based on Silver (Ag), gold (Au) and zinc (Zn) nanoparticles and nanoparticle bases drug delivery systems have been found effective against many viral infections like HIV, HSV, HCV, monkey pox virus and zika virus [166]. Currently under clinical trial nanobodies includes BGB-DXP592 (US clinical trial # NCT04551898), LY3819252 (US clinical trial #NCT04497987), REGN10933/REGN10987 monoclonal antibodies (US clinical trial # NCT04426695) and antibody fragments (INOSARS) (US clinical trial # NCT04514302). Dexamethasone, which has been recommended to treat COVID-19 patients (https://www.recoverytrial.net/results), nano-formulations have been proposed to be more effective [167]. In addition to the two approved vaccines i.e. mRNA-1273 from Moderna and BNT162b2 from Pfizer – BioNtech, many other vaccines under clinical trials have been lipid nanoparticle–formulated (Fig. 5) [168].

While scientists around the world are working on the development of effective therapeutics and vaccines against COVID-19. Further research studies are needed to understand the SARS-CoV-2 infections in humans and the zoonotic transmission of CoVs through clinical manifestations and study these viruses in detail. On the other hand, the pandemic’s catastrophic economic impact is pushing governments to reopen their economies, and this creates a public health quandary. Currently, the option is to minimize viral transmission through social distancing and efficient public health policy.

## Funding

We thank Higher Education Commission (HEC), Pakistan and School of Biological Sciences, University of the Punjab, Lahore, Pakistan for providing funding and research facilities for this work.

## Declaration of Competing Interest

The authors declare that they have no known competing financial interests or personal relationships that could have appeared to influence the work reported in this paper.
